# Correction to “A Comparison of the Biochemical Modifications Caused by Toxic and Non‐Toxic Protein Oligomers in Cells”

**DOI:** 10.1111/jcmm.70708

**Published:** 2025-07-17

M. Zampagni, R. Cascella, F. Casamenti, C. Grossi, E. Evangelisti, D. Wright, M. Becatti, G. Liguri, B. Mannini, S. Campioni, F. Chiti, and C. Cecchi, “A Comparison of the Biochemical Modifications Caused by Toxic and Non‐Toxic Protein Oligomers in Cells,” *Journal of Cellular and Molecular Medicine* 15, no. 10 (2011): 2106–2116, https://doi.org/10.1111/j.1582‐4934.2010.01239.x.

In the article, duplicated image panels between this (Figure 3A) and another article [18] published previously by an overlapping group of authors. The authors confirmed that these are indeed identical images, depicting the same experimental condition used in both studies, yet the first published figure was not correctly referenced. The authors apologize for this oversight.

The corrected caption of Figure 3 is below:

**Figure 3**

Representative confocal microscope images showing intracellular Ca^2+^ levels in SH‐SY5Y (**A**) and Hend (**B**) cells exposed for the indicated time lengths to oligomers formed under conditions A or B. The figure also shows images obtained with untreated cells, cells exposed to the native protein and cells exposed to type A oligomers in a Ca^2+^‐free medium or following a 24 h pre‐incubation with vitamin E. After oligomer exposure cells were treated with Fluo‐3‐AM. Kinetic plots showing the fluorescence associated with intracellular Ca^2+^ versus time elapsed after oligomer addition to the SH‐SY5Y and Hend cell media, after subtracting the fluorescence measured for untreated cells. A variable number of cells ranging from 20 to 30 were analysed for every time point in three different experiments. Figure 3A Condition A and B – 60 min panels, as well as the Untreated – 0 min and the Native HypF‐N – 60 min panels were reproduced from the previous work of Campioni et al. [18].

**Reference**

[18] S. Campioni, B. Mannini, M. Zampagni, A. Pensalfini, C. Parrini, E. Evangelisti, A. Relini, M. Stefani, C. M. Dobson, C. Cecchi, and F. Chiti, “A Causative Link Between the Structure of Aberrant Protein Oligomers and Their Toxicity,” *Nature Chemical Biology* 6, no. 2 (2010): 140–147, https://doi.org/10.1038/nchembio.283.

